# Biliary atresia: pathology, etiology and pathogenesis

**DOI:** 10.2144/fsoa-2019-0153

**Published:** 2020-03-17

**Authors:** Mukul Vij, Mohamed Rela

**Affiliations:** 1 Senior Consultant Histopathologist, Department of Pathology, Dr Rela Institute & Medical Centre, Chennai, Tamil Nadu, India, 600044; 2 Institute of Liver Disease & Transplantation, Chairmen, Dr Rela Institute & Medical Centre, Chennai, Tamil Nadu, India, 600044; 3 Liver Transplant Unit, Kings College Hospital, London SE5 9RS, UK

**Keywords:** biliary atresia, etiology, genetics, immunology, morphogenesis, pathogenesis, pathology, toxins, viruses

## Abstract

Biliary atresia is a progressive fibrosing obstructive cholangiopathy of the intrahepatic and extrahepatic biliary system, resulting in obstruction of bile flow and neonatal jaundice. Histopathological findings in liver biopsies include the expansion of the portal tracts, with edematous fibroplasia and bile ductular proliferation, with bile plugs in duct lumen. Lobular morphological features may include variable multinucleate giant cells, bilirubinostasis and hemopoiesis. The etiopathogenesis of biliary atresia is multifactorial and multiple pathomechanisms have been proposed. Experimental and clinical studies have suggested that viral infection initiates biliary epithelium destruction and release of antigens that trigger a Th1 immune response, which leads to further injury of the bile duct, resulting in inflammation and obstructive scarring of the biliary tree. It has also been postulated that biliary atresia is caused by a defect in the normal remodelling process. Genetic predisposition has also been proposed as a factor for the development of biliary atresia.

## Lay abstract

Biliary atresia is an obliterative fibrosing cholangiopathy of the intrahepatic and extrahepatic bile ducts. Even after successful surgery (Kasai portoenterostomy), most patients are likely to show progressive liver dysfunction. It is the commonest indication for liver transplantation during childhood. Liver biopsies demonstrate features of large duct obstructive cholangiopathy. Several theories have been proposed for etiopathogenesis including viral infection, immune-mediated bile duct injury and genetics. Herein, we review the pathology and etiopathogenesis.

Biliary atresia (BA) is a progressive fibrosing obstructive cholangiopathy involving both the extrahepatic and intrahepatic biliary system [1]. Clinical presentation is characterized by direct or conjugated hyperbilirubinemia, acholic stool, dark urine, variable levels of hepatosplenomegaly and progressive hepatic failure [2]. If untreated, affected infants develop rapidly progressing fibrosis, leading to portal hypertension and end-stage liver disease, invariably resulting in death within the first 2 years of life. Early diagnosis is critical so that surgical intervention, also known as Kasai portoenterostomy (KPE), can remove the atretic extrahepatic biliary tree in an attempt to re-establish the biliary flow to the intestine by creating a Rouxen-Y intestinal conduit [3]. This can help prevent worsening of the liver disease and delays the need for liver transplant if performed in the first 2–3 months of life, with best results at approximately 30–45 days old [4,5]. Overall, KPE is associated with short-term jaundice clearance in 50–60% of infants in western population, with successful biliary drainage, which manifests as yellow colored stool, usually resolving within the first postoperative week [6]. When KPE is performed within the first 60 days, 70–80% of patients show early jaundice clearance. If performed between 60 and 90 days, 40–50% of patients show jaundice clearance and after 90 days of age only 25% of patients show early jaundice clearance [7]. Approximately a third of the patients can survive with native liver at the age of 10 years and a fourth at age 20 years [6]. In a Japanese study, the rate of early jaundice clearance was 57% and the 10-year survival rate with their native liver was 54% [8]. Various studies from India have reported 36.2–47.2% of early jaundice clearance [9–11]. Our group reported successful KPE in a 29.4% of children over 90 days of age and concluded that in the absence of synthetic liver failure, age should not be a disqualification for performing KPE [12].

Over the years, several factors have been proposed to predict post-KPE outcome. Poor outcomes have been associated with late diagnosis and delayed KPE, presence of associated extrahepatic anomalies such as biliary atresia splenic malformation (BASM), portoenterostomy performed by laparoscopic method, failure to achieve jaundice clearance in 3 months post-KPE and repeated cholangitis [13]. A serum direct bilirubin of less than 2.0 mg/dl within 3 months post-KPE, is highly predictive of good outcome with one’s native liver [14]. Five-year survival rate without liver transplantation (LT) following KPE is also determined by the number of procedures performed by the medical center. Hospitals performing more than five cases per year had better surgical outcomes as when compared with hospitals performing a smaller number of KPE [15]. A study from France reported significant differences in overall survival at 4 years if KPE was done at a center with a higher caseload [16]. Unfortunately, even with KPE, significant progression of fibrosis and development of cirrhosis occurs in the majority of patients requiring liver transplant [17]. BA is the leading indication for LT in children and, to date, no other medical treatments have been identified.

## Epidemiology

BA is the most common cause of neonatal jaundice, representing approximately 25–30% of cases and has remained unchanged over the years. There is regional variability in the reported incidence of BA, with a higher incidence being recorded in Asia and the Pacific region. The disease is diagnosed in approximately 1 per 5000 in Taiwan, 1 per 10,000 in Japan, 1 per 17,000–19,000 live births in UK and France, 1 in 19,000 in the Netherlands and 1 per 15,000 live births in the USA [18–23]. Higher incidence is also reported within Hawaii, which is an island surrounded by the Pacific Ocean [24]. Higher incidences are also seen among Inuits and Native Americans [25].

A recent report suggests that this difference in incidence of BA correlates with the frequency of the most prevalent human leukocyte antigen (HLA) haplotype frequency, suggesting that the incidence is related to the human colonization [26]. Immunological homogeneity that is preserved in a given ethnicity is more likely to lead maternal microchimerism and may reasonably explain this genetic predisposition. Females are affected slightly more frequently than males [17]. Few studies have suggested both seasonal variability in incidence, as well as regional clustering of cases [21,27–29], however, a large study of 119 Japanese infants did not demonstrate any evidence of seasonal clustering [30]. Familial clusterings are exceedingly rare and disease concordance in twins is unusual [31–36]. The reason for this is unclear, but suggests that environmental or genetic factors may contribute to syndromic BA incidence.

### Clinical phenotypes

BA has three major clinical phenotypes. The most common forms are isolated, perinatal, acquired or nonsyndromic and account for 70–80% of cases [37]. The fetal/embryonal, syndromic or congenital form is associated with extrahepatic developmental anomalies (including asplenia, polysplenia, double spleen, cardiovascular defects, situs inversus, intestinal malrotation, small-intestinal atresia, pancreaticobiliary maljunction and various positional abnormalities of the portal vein and hepatic artery) and is referred to as BASM syndrome, which accounts for 10% of cases [37,38].

In syndromic type, clear racial differences have been reported, and is infrequent in the high incidence areas of the world for BA (e.g., Japan and Korea). One study determined that syndromic BA occurred significantly less frequently in China compared with the Western world [39]. In a series from India, 6% of cases had BASM [40]. In syndromic cases, it appears to be due to a primary failure of extrahepatic bile duct development, the gallbladder is invariably atrophic and the common bile duct (CBD) absent. Infants with BASM are usually girls and they have a higher association with maternal diabetes mellitus and thyrotoxicosis than those with the nonsyndromic form and might have a worse outcome after hepatoportoenterostomy.

In a French series, 50% of infants with BASM had CFC-1 mutations [41]. This gene, present at Ch2q 21.1, encodes for CRYPTIC protein, which is involved in signaling during embryonic development and mutations have been related to defects in organ development including heterotaxy syndromes and congenital heart diseases, such as transposition of the great vessels/double outlet right ventricle. The genetic syndromes that have been associated with BA include cat-eye syndrome, Mitchell–Riley syndrome, Zimmermann–Laband syndrome and Fanconi anemia complementation group Q [42]. Some infants with BA have nonsyndromic congenital anomalies such as esophageal atresia, jejunal atresia, anorectal atresia, etc. for which no convincing genetic explanation is available. A small percentage of infants with BA have injury to the biliary tree, which produces cystic dilation that resembles choledochal cyst [43,44]. Rarely, the cystic change is confined to intrahepatic ducts. This type of BA is now called as ‘cystic’ BA (formerly called ‘correctable’ BA), which comprises 5–10% of patients [43,45] and can be identified on antenatal sonography of the fetus in approximately 40% of affected cases. BA is also classified based on the level of extrahepatic obstruction of the biliary tree. In type 1 BA (approximately 5%), obstruction is at the level of the common bile duct. In type 2 BA (approximately 2%), atresia occurs at the level of the common hepatic duct. In type 3 BA (90%), there is atresia at porta hepatis [17].

### Pathology

Liver biopsy is the cornerstone for the diagnostic work-up of infants with neonatal cholestasis. It is standard practice in most pediatric hospitals to obtain a percutaneous tru-cut liver biopsy before any surgical procedure [46]. Multiple studies have reported utility of liver biopsies in cases of neonatal jaundice. A liver biopsy can correctly predict obstruction in more than 90% of cases, with a reported accuracy rate ranging between 60 and 95% [46,47]. This variation may reflect pathologist’s expertise, or this may be related to liver biopsy timing, however, it is still unclear. An adequate liver biopsy should be at least 2.0 cm long, 0.2 mm wide and not fragmented and ideally contain ten portal areas [46]. If it is a surgical wedge, it should be sufficiently deep to include six complete portal tracts independent of the liver capsule. The pathologist’s main role lies in recognizing morphological features of large duct obstructive cholangiopathy, and BA is by far the most frequent cause of large duct obstruction in the neonate and infant.

**Figure 1.**
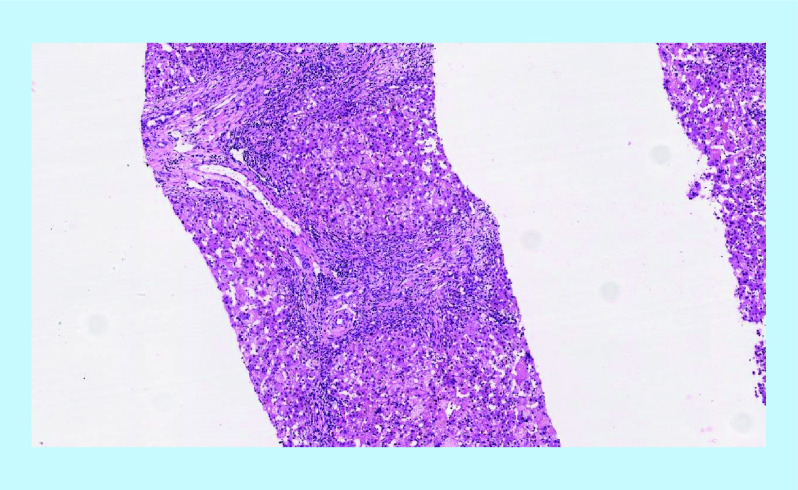
Liver biopsy displaying portal fibrous expansion with ductular proliferation (hematoxylin and eosin, x10).

The key histopathological findings for the diagnosis of BA are present in the portal tracts [46–48]. Portal findings in BA simulates those of large-duct obstructive cholangiopathy due to any other etiologies. Portal tracts show expansion and demonstrate edematous fibroplasia with bile ductular proliferation, consisting of anastomosing ductules located at the periphery of the portal tracts (Figure 1). This finding represents the most consistent indicator of the presence of a biliary obstructive process [48]. Mild degrees of ductular reaction may also be seen in neonatal hepatitis of various etiologies. Ductular cholestasis (inspissated bile plugs within ductular lumen) is commonly seen and represent a useful diagnostic feature. Duct/ductal bile plugs and portal stromal edema are strong histologic predictors of BA. Proliferation of fibroblasts, as well as a variable amount of inflammatory cells, especially neutrophils are usually present. When neutrophils are observed within the lumen of the ducts, a superimposed cholangitis should be suspected. Portal tracts may also show hemopoietic elements especially myelopoiesis. Although disease progression evolves with age, biopsy findings of large duct obstructive cholangiopathy are present in most cases of BA, even for biopsies performed at less than 30 days of age [48]. Some patients may still show nonspecific histologic findings in early biopsies and may result in a false negative diagnosis. Azar *et al.* described four patients with neonatal conjugated hyperbilirubinemia, in whom, the initial liver biopsy demonstrated no significant bile ductular proliferation, despite subsequent development of BA [49]. Initial biopsies demonstrated paucity of interlobular bile duct in three infants, with a bile duct to portal tract ratio of 0.3–0.4 (normal: 0.9–1.8). The fourth infant showed a normal bile duct to portal tract ratio of 1.0. Subsequent biopsies performed at 9–12 weeks of life demonstrated ductular proliferation and BA was noted at the time of a KPE. The fourth child underwent liver transplant, with the explant demonstrating biliary pattern cirrhosis. False-positive diagnosis of BA based on cholangiographic findings are also documented in the literature [50,51].

**Figure 2.**
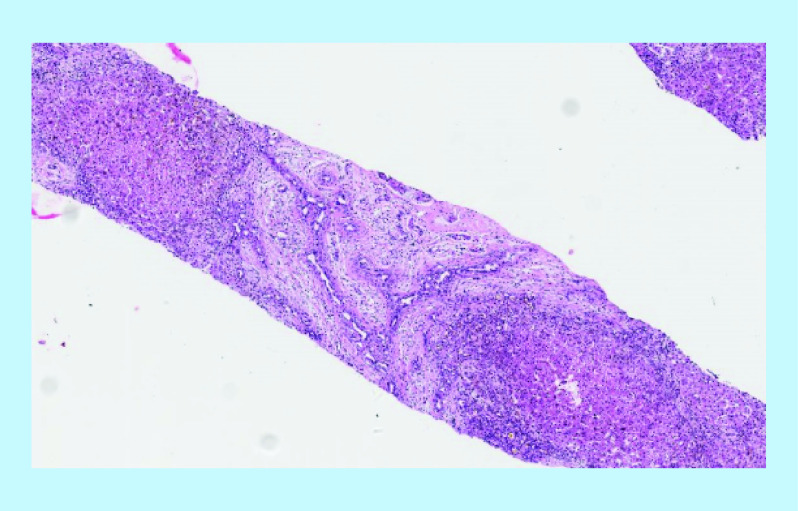
Ductal plate malformation like arrays in atresia biopsy (hematoxylin and eosin, x10).

The presence of circumferential biliary ductular structures within the connective tissue of portal tract in BA, which resemble primitive ductal structures or ductal plate malformation (DPM), referred to as ‘DPM-like arrays’ may also be seen (Figure 2). The intrahepatic bile ducts develop from the fetal ductal plate through an orderly process of selection and apoptosis called ductal plate remodeling [1]. Failure of the remodeling process of the ductal plates during embryogenesis are believed to give rise to DPM, in which intrahepatic bile ducts retain the fetal configuration. The persistence of this developmental configuration of the fetal intrahepatic biliary tree on liver histology in BA patients has led to the suggestion that DPM could be a marker for abnormal morphogenesis in BASM and, by extension, an indicator of unfavorable prognosis. However, DPM has been identified in both embryonic and perinatal form of BA by various studies [52]. In BA biopsies, both definite as well as possible DPM-like configurations have been described [53]. Definite DPM-like arrays are peripheral, circumferential with a central conspicuous fibrovascular core. Possible DPM-like arrays are smaller and compact around a small mesenchymal core with inconspicuous vasculature. The overall incidence of DPM reported in most series is 20–50% [1,53–57]. Lobular features include variable degrees of multinucleate giant cell transformation of hepatocytes, extramedullary normopoiesis, myelopoiesis and megakaryopoiesis, cellular ballooning, hepatocanalicular bilirubinostasis with rosetting and patchy inflammation with spotty necrosis. Periportal copper deposits are seen in most liver biopsy specimens within first 4 months, but it does not differentiate extrahepatic from intrahepatic causes of pediatric cholestatic disorders.

Initially, extrahepatic biliary tract injury and fibrous obliteration represent the chief finding in BA. Destructive injury of smaller intrahepatic bile ducts is thought to occur later in the course of the disease. Ductopenia represents an important factor, which leads to the progression of liver dysfunction that occurs in a significant proportion of cases and therefore eventually results in liver failure and the need for transplant. Lymphocytic cholangitis, as well as signs of duct epithelial injury, including nuclear enlargement, loss of nuclear polarity, nuclear stratification and vacuolated cytoplasm, are often seen in these cases, followed by destruction of bile ducts. Mild lymphocytic inflammation is usually present within portal tracts in BA. Other inflammatory cells, including eosinophils, plasma cells and macrophages, are also present. In addition to its role in diagnosis, evaluation of the liver biopsy may also reveal prognostically significant histologic features. Higher stages of portal fibrosis have been associated with more severe portal hypertension and increased risk of LT [58]. Pape *et al.* studied picrosirius red staining in combination with computerized morphometry to examine BA liver biopsies and calculated a score (Vfib), which characterized the mean volume of fibrosis [59]. Liver transplant-free survival was significantly higher in those patients with Vfib of less than 2.5%. However, in some studies there was no correlation between the degree of fibrosis and survival of native liver [60,61].

The transformation and proliferation of hepatic stellate cells (HSCs) from fat-storing cells to myofibroblasts in BA plays an important role in hepatic fibrogenesis. HSCs are responsible for increased extracellular matrix, which leads to progressive scarring [62]. Expression of an intracellular microfilament protein named α-smooth muscle actin (α-SMA) determines the HSC activity. Shteyer *et al.* performed α-SMA immunohistochemistry in liver biopsies and studied its expression in the portal and lobular areas. The study reported that degree of α-SMA expression and fibrosis scores predict the outcome of KPE, such as jaundice clearance and native liver survival [63]. A reduction in conjugated bilirubin 3 months after KPE was negatively correlated with α-SMA expression in a study by Dong *et al.* [64]. Kinugasa, *et al.* examined ductular proliferation in biopsies by immunohistochemistry using the antibody anticytokeratin 7 (CK7) [65]. The number of CK7-positive cells were counted within the 40-microm-thick interstitium, along the limiting plate, and the CK7-positive cell number per unit length of the limiting plate was estimated. Cases with poor bile drainage after KPE had a higher number of CK7-positive cells.

The impact of DPM on BA prognosis has also been studied. Some studies have reported that presence of DPM had worse prognosis and there is poor bile flow after KPE in infants with BA [56]. Arii *et al.* reported higher bilirubin post-KPE in the presence of DPM [57]. However, the incidence of long-term complications and the necessity for LT was not influenced by the presence of DPM in their study. Our study demonstrated that the morphologic presence of DPM had an impact on survival of native liver in patients with synthetic liver failure who underwent liver transplant after a prior KPE or underwent primary LT because of delayed diagnosis of BA [1]. Children with DPM, who had a prior KPE, underwent liver transplant approximately 14 months earlier to children without DPM.

### Differential diagnosis

Differential diagnosis of diseases with features of obstruction on a liver biopsy includes total parenteral nutrition (TPN)-related hepatopathy, choledochal cyst, Alagille syndrome, α1-antitrypsin deficiency, multidrug resistance protein 3 MDR3 deficiency, cystic fibrosis, neonatal sclerosing cholangitis (NSC) or any other cause of mechanical obstruction [47,66]. Ductal/ductular proliferation can be seen in A1AT deficiency in the neonates. Also, Periodic-acid Schiff after diastase positive intracytoplasmic globules are generally not as prominent in neonates as in the older patient. Hypoplastic extrahepatic ducts, defined as abnormally small but patent ducts associated with Alagille syndrome, may result in confusion with BA. Though liver biopsies in Alagille syndrome typically reveal paucity of interlobular bile ducts, bile ductular proliferation suggestive of obstruction, can be noted early in the course of the disease. MDR 3 deficiency is caused by genetic defects in *ABCB4* gene encoding MDR3 protein. MDR3 causes cholangiopathy with ductular proliferation and bile plugs [67]. It usually presents in older infants. Immunostaining with MDR3 and genetic studies will help to confirm the diagnosis. History of TPN therapy should always be provided to the pathologist. The liver in patients with cystic fibrosis demonstrates steatosis and eosinophilic mucus plugs in bile ducts. Cholangiography in the case of NSC highlights patent extrahepatic bile ducts. This. in contrast to BA, demonstrates thin and irregular appearing extra- and intrahepatic bile ducts. NSC is associated with extra hepatic clinical manifestations such as ichthyosis and scarring alopecia. We have also encountered rare cases of fibropolycystic disease in early infancy clinically mimicking BA (unpublished data).

### Morphological findings in the resected extrahepatic biliary tree remnants

The destructive fibroinflammatory process that underlies BA, may involve a short segment of a duct, an entire duct or the entire biliary system. Multiple studies have described morphology of the resected biliary remnant [68–71]. Gautier and Eliot classified the biliary remnants into three types: type 1, completely atretic duct with no lumen; type 2, multiple small ducts having Lumina less than 50 μm, arrayed around a fibroinflammatory cord; and type 3, duct with central lumen, sometimes containing bile with epithelial injury and degenerative changes. Squamous metaplasia of the biliary channels may be observed.

Conflicting results have been reported from various studies, which have analyzed duct diameter and number of bile ducts and establishment of bile flow after KPE. Few investigators believed that bile flow is most likely to occur when the diameter of the residual ducts exceeds 150 μm [70–72]. However, another study of the extrahepatic biliary remnants of 205 cases of BA determined that the patterns of bile duct obliteration are not indicative of prognosis [69]. Occasionally, nodules of cartilage have been noted at the porta [73] and squamous metaplasia has been described.

Cystic BA is a rare cystic expansion of atretic extrahepatic bile duct remnants in young infants, which terminates blindly at the distal end [43]. Cystic BA cysts typically lack epithelium and inflammation. There is subepithelial zone of dense paucicellular cicatricial layer, which is associated with a zone of myofibroblastic hyperplasia and involves the inner and/or outer layer of this laminated collagen. There is shedding of this layer forming inner septa in a complex cyst. Gall bladder may show atresia or normal appearance.

### Liver explants

The explanted livers in most BA patients are firm, greenish and micronodular. Biliary pattern cirrhosis with irregular nodules are observed on microscopy. Explants, especially in older children and adults, may show two distinct areas, central hypertrophic segments and atrophic lateral segments. The segments of the liver where bile drainage is well maintained may show regeneration, while other segments without sufficient bile drainage show atrophy [74]. Portal areas demonstrate prominent arterioles and attenuated veins. Progressive loss of intrahepatic bile ducts is identified, in untreated cases, as early as 5–6 months and occurs in most patients despite treatment, even when adequate biliary drainage is established [75]. Bile duct loss is heterogenous and large nodules with preserved architecture and ducts reflect local differences in biliary flow [76]. Dilated large ducts, with patchy to extensive ulceration of lining epithelium and with bile impregnation of wall and biliary sludge in lumen, can be seen. Bile lakes with fibrous wall and xanthogranulomatous inflammatory reaction composed of sheets of pigment laden histiocytes, admixed with multinucleated giant cells can also be seen. Multiple theories have been proposed for the formation of bile cysts and bile lakes in patients with BA. Some authors suggest that formation of biliary cyst is secondary to the intrahepatic fibro-obliterative process leading to ulceration and erosion of the lining epithelium of the biliary radicals resulting in leakage of bile [77–79]. Other authors suggest that the ongoing inflammatory process in the portal tracts results in obstruction of intrahepatic biliary radicals, which causes cholangitis and bile cysts formation. Some studies hypothesized that presence of DPM lead to bile cyst formation. The bile duct injury is more in the presence of DPM. Although the incidence of DPM was high in our series, it also correlated well with the occurrence of bile cysts and bile lakes. This is suggestive of a more exaggerated bile duct injury in the presence of DPM [1]. There are deposits of copper/copper associated proteins in periportal and periseptal hepatocytes. Mallory Denk bodies may also be seen. Rarely hepatocellular carcinoma has been identified in the explanted liver specimen [80].

## Etiology & pathogenesis

The etiopathogenesis of BA is multifactorial and has been subject of intense investigation, with a number of possible pathomechanisms being proposed. These can be broadly categorized as viral infections, toxins, genetic variants, immunogenic abnormalities or autoimmune disorders, maternal microchimerism, vascular disturbances and defects in morphogenesis.

### Viral infection

The seasonal variation of BA cases reported in some studies, the absence of a significant hereditary component and the experimental evidence of virus-induced BA in animal models, have suggested a role of viral infections in human cases of BA. An initial bile duct injury caused by viral infection would lead to a progressive immune-mediated fibro-obliterative process, resulting in damage and destruction of the bile ducts. In 1974, Benjamin Landing first proposed that viral infection of the liver and hepatobiliary tree causes BA and other infantile obstructive cholangiopathies [81]. The possible role of several viruses has been suggested in this setting, including cytomegalovirus (CMV), rheovirus, human herpes virus, human papillomavirus, adenovirus, Epstein–Barr virus, hepatitis B virus, parvovirus B19 and rotavirus (RV). Reovirus, CMV and RV have been studied extensively.

Reovirus is a double-stranded RNA virus from the Reoviridae family. Reovirus 3 was suggested as a cause of BA and neonatal hepatitis on the basis of clinical and experimental studies. Tyler *et al.* detected reovirus RNA from hepatobiliary tissues of 55% of patients with BA and 78% of patients with choledochal cysts [82]. This was much higher when compared with infants with neonatal cholestasis from other causes. However, this association was questioned by other investigators [83,84]. Few studies reported an increased prevalence of anti-reovirus IgG and IgM antibodies in BA infants when compared with controls [85,86]. However, other studies were not able to confirm such results [87,88]. No convincing association between BA and infection with common hepatotropic viruses, in other words, hepatitis A, B and C, exists in the literature. Riepenhoff *et al.* reported RV DNA in ten liver samples from infants with BA or choledochal cyst [89]. However, more recently a large scale multicenter study from North America analyzed the prevalence of acute asymptomatic group A and C RV (RV-A and RV-C) infection in neonates with cholestasis, <6 months old, among which were 40 infants with BA [90]. They concluded that RV infection in the first 6 months of life is common in cholestatic infants of any cause and infection could have different pathogenetic effects by initiating different hepatic immunologic reaction in infants with versus without BA, or could lack pathogenetic significance.

A study conducted by researchers at Kings College Hospital (London, UK), defined a group of infants with BA who have IgM antibodies to CMV [91]. This group included 10% infants of their clinical series and were more commonly seen in infants from non-Caucasian parents. Clinically, these infants were older at the time of diagnosis and came late to surgical intervention. Biochemically, these infants had higher bilirubin and AST levels, with larger spleens as measured on ultrasonography than comparable IgM-ve BA infants, even when corrected for their older age. IgM+ve BA was characterized by a greater degree of inflammation and fibrosis. More lobular cholestasis was identified in infants with CMV IgM-ve isolated BA. CMV IgM+ve BA had a poorer outcome, with a reduced jaundice clearance, native liver survival and increased mortality. Interestingly no liver biopsies in their clinical series stained positive for CMV immunostaining. In a recent study of Chinese infant CMVs, DNA and/or protein was identified in the livers of 51 infants and a second study of a smaller cohort, determined a low percentage of patients with normalization of bilirubin levels after hepatoportoenterostomy [92,93]. CMV can infect biliary epithelia, as demonstrated by CMV inclusion bodies. CMV has been implicated in neonatal hepatitis, paucity of intralobular bile ducts and ischemic vasculopathy. It has been suggested that the CMV infection is short-lived, leading to an inability to identify the virus in some cases and that viral infection of cholangiocytes sets the stage for an altered autoimmune response targeting cholangiocytes, which leads to progressive biliary tract injury and fibrosis. One limiting factor in viral identification includes the various type of methodologies used to detect infection. Another potential factor is the ability of the neonatal immune system to clear the virus [94]. In an experimental mouse model of BA, RV was effectively cleared from the hepatobiliary system by the second week of infection. The theory of a viral origin of BA is also supported by the observation that most livers of BA patients are immunopositive for Mx protein, which is a strong indicator of previous or active viral infections [95].

One study tested 74 liver biopsies (taken during KPE) by PCR for viral DNA (human herpes virus, Epstein–Barr virus, varicella zoster virus, CMV, adenovirus, parvovirus B19 and polyoma BK and RNA viruses [enteroviruses, RV and reovirus 3]) [96]. Immunostaining for Mx protein was also performed. Viral DNA/RNA was found in less than half of the biopsies (8/74 CMV, 1/74 adenovirus; 21/ 64 reovirus, 1/64 enterovirus). Co-viral infection was detected in few. 92% of the liver biopsies demonstrated immunohistochemical expression of Mx proteins in hepatocytes and bile duct epithelial cells, which could suggest that the known hepatotropic viruses do not play a major role in the etiology and progression of BA. Their incidence in a review of studies that analyzed human samples via PCR experiments concluded that although a considerable number of PCR studies have sought to clarify a viral role in the pathogenesis of BA using human samples, the findings have been contradictory and have not succeeded in achieving an obvious differentiation between causative and accidental infection of the focused virus [97].

## Immunologic injury

The histologic evidence of lymphomononuclear inflammatory cells infiltrating the vicinity of injured interlobular bile ducts and within the duct epithelium, suggests that in BA immunologic mechanisms play an important role in bile duct damage [52]. An initial viral or toxic insult to the biliary epithelium leads to newly expressed or altered antigens on the surface of bile duct epithelium. These are then presented by macrophages to naive T lymphocytes. Primed Th1 lymphocytes then organize an immune response by releasing proinflammatory cytokines and recruiting cytotoxic T cells, ultimately causing bile duct epithelial injury, eventually resulting in scarring and obliteration of the bile duct.

Mack *et al.* obtained frozen liver tissue from patients with BA, choledochal cysts, neonatal giant cell hepatitis, TPN-related cholestasis and normal control subjects [98]. Immunohistochemistry with CD8, CD4 T cells and histiocytes (CD68) demonstrated increased portal inflammatory cell infiltrates in BA. Reverse transcription-PCR analysis of BA tissue demonstrated a Th1-type cytokine profile with expression of IL-2, IFN-γ, TNF-α and IL-12, in contrast to normal liver controls, choledochal cyst or neonatal giant cell hepatitis. The researchers suggested that there is a distinctive portal inflammatory environment in BA, involving CD4 Th1 cell-mediated immunity. Other pediatric cholestatic diseases did not demonstrate similar inflammation. This suggests that inflammation in BA is not a secondary response to cholestasis, but rather indicates a specific immune response involved in its pathogenesis.

Zhang *et el.* studied the infiltration of CD4+T cell subsets and their clinical significance in BA [99]. BA liver samples were collected at the time of surgery and were divided into good (BA1, n = 16) and poor prognosis (BA2, n = 14). Control samples were from patients with choledochal cyst (n = 8). By utilizing multiplex immunohistochemistry, the researchers evaluated the infiltration level of CD4+T cell subsets in the portal areas. Quantitative reverse transcription-polymerase chain reaction (RT-qPCR) and flow cytometry studies were further applied to evaluate regulatory T cells (Tregs) subsets. They demonstrated that hepatic infiltration of Th1, Th2, Th17 and ICOS+Treg cells were significantly increased in BA patients compared with controls and was negatively associated with prognosis, while high infiltrating ICOS−Tregs showed a favorable outcome.

Shinkai *et al.* performed quantitative rt-PCR to identify the inflammation by quantifying lymphocyte density and expression of TH1 cytokines such as IL-2 and TNF-α in liver biopsies with patients of BA [100]. They demonstrated significant portal increase in CD8+ lymphocytes in liver biopsy specimens of infants with BA. Also, IL2 mRNA expression was significantly elevated within the BA livers compared with other cholestatic disorders.

Narayanaswamy *et al.* investigated the inflammatory components by studying a panel of cellular adhesion molecules (sICAM-1, sVCAM-1) and soluble proinflammatory mediators (T helper 1 [IL-2 and interferon] and T helper 2 [IL-4 and IL-10]) cytokines and macrophage markers (TNF and IL-18) in 21 consecutive infants with BA post-KP [101]. The authors concluded early circulating inflammatory process in BA is persistent, progressive and involves a nonpolarized T cell, histiocyte and cell adhesion molecule response, only partially ameliorated by KP. A reduction in the expression of the histiocyte marker (CD68) within the liver and biliary remnants and, reduction of ICAM-1 expression on infiltrating cells in the biliary remnants, appears to be associated with a better postoperative prognosis.

Inappropriate host immunological reactions against unknown ligands via the Toll-like receptor (TLR) cascades, may trigger progressive inflammatory destruction of the bile ducts that manifests as BA in infants. Saito *et al.* reported upregulation of TLR8 mRNA, encoding the receptor for single-stranded RNA in livers from BA patients at diagnosis compared with non-BA patients [102]. They also demonstrated significantly higher mRNA expression of TLRs 3 and 7 at diagnosis in those BA patients who required liver transplant when compared with the nonliver transplant group. Huang *et al.* also analyzed hepatic RNA from BA patients and demonstrated significantly higher levels of TLR7 when compared with choledochal cyst patients [103]. Immunostaining determined that there was a strong expression of TLR7 in bile duct epithelia, nonparenchymal cells and neutrophils. Shivakumar *et al.* also investigated the role of the innate immune response in the pathogenesis of BA [104]. Using a model of RV-induced BA in newborn mice, they determined that activated NK cells were the most abundant cells in extrahepatic bile ducts at the time of obstruction. Depletion of NK cells prevented the injury of the biliary epithelium, as well as maintained luminal continuity between the liver and duodenum and enabled bile flow, after RV infection.

Osteopontin is a Th1 cytokine that is secreted by a variety of cell types and plays a key role in recruitment of immune cell to inflammation sites, as well as extracellular matrix formation and fibrosis. Bezerra *et al.* reported upregulation of proinflammatory genes including IFN-γ and osteopontin [105]. Whitington *et al.* demonstrated increased levels of osteopontin in BA liver tissue that localized to bile duct epithelia and correlated with bile duct proliferation and fibrosis [106].

The Tregs are a subset of CD4+ T cells that have role in suppressing the immune response, thereby maintaining homeostasis and self-tolerance. Tregs are able to inhibit T-cell proliferation, cytokine production and play a critical role in preventing autoimmunity. In neonates, the percentage of Tregs increases significantly in the first 5 days of life, reaching adult levels at that time. In BA, deficits in the number or function of Tregs would allow for inflammation to flourish unchecked. Brindley *et al.* investigated the quantitative changes in Tregs in the presence of viral infection in infants with BA [107]. When compared with controls, significant deficits in Treg frequencies were identified in the peripheral blood of BA patients. There were also marked deficits in those BA patients who were CMV positive. The authors suggested that loss of adequate numbers of Tregs in BA would result in decreased inhibition of inflammation or autoreactivity, potentially allowing for exaggerated bile duct injury. Shivakumar *et al.* suggested IFN-γ-driven bile ducts obstruction is a key pathogenic mechanism of disease [108]. In neonatal mice infected with RV, the genetic loss of IFN-γ suppressed the tissue-specific targeting of T lymphocytes and completely prevented the fibroinflammatory extrahepatic bile duct obstruction, resulting in resolution of jaundice more than 80% improvement in long-term survival. Loss of IL-12 has not been shown to modify the progression of bile duct obstruction in experimental BA [109]. Klemann *et al.* demonstrated upregulation of the IL17 axis in BA and its experimental model. IL17 inhibition improved the clinical outcome and liver inflammation in the murine model. They conclude that IL17-producing γδ T cells are a causative factor in the inflammatory destruction of the biliary tree in experimental BA [110]. IL-8 is expressed and secreted at high levels both *in vitro* and *in vivo* in human BA, with recent studies suggesting that it is critical for BA inflammation and disease progression [111].

### Environmental toxins

Outbreaks of BA in lambs and calves in New South Wales, Australia related to ingestion of toxin-containing plants by pregnant animals during drought conditions, as well as the reported clustering of BA cases in humans, have led to the proposal that an environmental toxin could be involved in BA pathogenesis [112]. Lorent *et al.* used a biliary secretion assay in zebrafish to isolate a previously undescribed isoflavonoid, biliatresone, from Dysphania species [113]. In zebrafish, this compound caused selective destruction of the extrahepatic biliary tree. The toxin also caused loss of cilia in a neonatal mouse extrahepatic cholangiocytes in culture and disturbance of cell polarity and monolayer integrity in cholangiocyte spheroids. Zinman *et al.* studied the cellular changes in mammalian cells caused by biliatresone that lead to BA. Mouse cholangiocytes were treated in 3D spheroid culture and neonatal extrahepatic duct explants with biliatresone and compounds that regulate glutathione [114]. They demonstrated that biliatresone decreases glutathione and SOX17 in mouse cholangiocytes. In 3D cell systems, this leads to cholangiocyte monolayer damage and increased permeability, in extrahepatic bile duct explants, it leads to disruption of the extrahepatic biliary tree and subepithelial fibrosis.

### Genetic influences

Considering the association of BA with DPM-like arrays, it has been suggested that a genetic mutation in the genes regulating bile duct development could act as a susceptibility factor or a modifier gene. One study investigated 102 cases of BA for Jagged 1 mutations, a Notch signaling ligand. Nine patients harbored a missense mutation in Jagged 1 [115]. Another study investigated the expression of Notch receptors in BA livers and determined that Notch 3 expression was increased in neovasculature and mesenchymal cells [116]. In addition, 10 % of BA patients have additional congenital defects related to abnormalities in left–right axis, suggesting that proteins regulating left–right patterning may be involved in the disease [117]. These include the cilium associated proteii inversin, cryptic protein (CFC1) and the zinc finger transcription factor ZIC3. BA like disorder have been reported in mice with a deletion of the Invs gene, however, no anomalies of biliary system have been described in humans with INVS mutations [118]. Two patients with BA and congenital heart defects had mutations in ZIC3 [119].

Leyva-Vega *et al.* screened a cohort of 35 BA patients for genetic deletions and duplications that might accord susceptibility to BA [120]. Two unrelated BA patients with overlapping heterozygous deletions of 2q37.3 were identified. Patient one had a 1.76 Mb (280 single nucleotide polymorphisms [SNP]), heterozygous deletion containing 30 genes. *AGXT* gene was sequenced, which is present only in liver, unlike the other 29 genes. The second patient had a 5.87 Mb (1346 SNP) heterozygous deletion containing 55 genes. Several other disorders including hypothyroidism, seizures, developmental delay and polysplenia were also identified. The overlapping 1.76 Mb deletion on chromosome 2q37.3 constitutes the critical region and the genes within this region could be candidates for susceptibility to BA.

Cui *et al.* evaluated the association of BA with germline copy number variants by comparing 61 BA cases and 5088 healthy individuals [121]. They reported a statistically significant increase in deletions at 2q37.3 in patients with BA. This resulted in heterozygous deletion of glypican 1 (*GPC1*), which encodes glypican 1, a heparan sulfate proteoglycan that regulates Hedgehog signaling and inflammation. Developmental biliary defects were produced in zebrafish with knock down of *GPC1*. Exposure of *GPC1* morphants to Cyclopamine, a hedgehog antagonist, partially rescued the GPC1-knockdown phenotype. Zebrafish injected with recombinant Sonic Hedgehog led to biliary defects similar to those of the *GPC1* morphants. Liver samples from patients with BA had reduced levels of apical *GPC1* in cholangiocytes when compared with samples from healthy individuals. Based on genetic analysis of patients with BA and zebrafish, *GPC1* appears to be a BA susceptibility gene. These findings also support a role for Hedgehog signaling in the pathogenesis of BA.

Garcia-Barcelo *et al.* performed a genome-wide association study using the Affymetrix 5.0 and 500 K marker sets to identify BA susceptibility loci [122]. The study demonstrated that the likelihood for developing BA is influenced by DNA variants in a region spanning 129 kb and encompassing the *XPNPEP1* and *ADD3* genes. *XPNPEP1* is expressed in epithelial cells of the hepatobiliary system. It is involved in the metabolism of inflammatory mediators. *ADD3* encodes ADDUCIN3, which is involved in the assembly of spectrin–actin network in erythrocytes and at sites of cell–cell contact in epithelial tissues, including that of the digestive tract, liver and biliary tract. DNA methylation is the only genetically programmed DNA modification process in mammals, which is involved in the regulation of several biological processes, including gene transcription, X-chromosome inactivation, genomic imprinting and chromatin modification. Sangkhathat *et al.* performed whole exome sequencing for 20 cases of BA to look for other infantile cholestatic disorders [123]. They identified 13 rare variants, which were detected in nine genes: four in *JAG1*, two in *MYO5B* and one each in *ABCC2, ABCB11, UG1A1, MLL2, RFX6, ERCC4* and *KCNH1*. They concluded that severe inflammatory cholangiopathy in BA might be a shared pathology among several infantile cholestatic syndromes.

One recent genetic study demonstrated intrahepatic biliary defects and upregulated hepatic expression of IFN-γ pathway genes, caused by inhibition of DNA methylation in zebrafish larvae. There was significant reduction of DNA methylation in bile duct epithelial cells from BA patients when compared with patients with other neonatal cholestatic diseases. This shows a possible etiologic link between decreased DNA methylation, activation of IFN-c signaling and biliary defects in patients [124]. MicroRNAs (miRNAs) are small noncoding RNA molecules that regulate gene expression through partial or complete complementarity with target mRNAs. The ability of serum miRNAs to distinguish BA from other forms of neonatal hyperbilirubinemia was examined by one study [125]. BA-specific serum miRNAs were identified using a microfluidic array platform and further validated in a larger, independent set of samples. miR-200b/429 cluster was significantly increased in the serum of patients with BA compared with patients with other hepatic cholestatic diseases. Bessho *et al.* investigated the biliary transcriptome to identify miRNAs with a potential role in the pathogenesis of bile duct obstructive cholangiopathy [126]. They identified 14 miRNAs whose expression was suppressed at the times of duct obstruction and BA.

HLA studies in BA might support a disease mechanism involving autoimmunity, but results to date are contradictory. One series reported that infants with nonsyndromic BA has a higher frequency of the HLA-B12 determinant, compared with both normal controls and to infants. BAs with syndromic haplotypes A9–B5 and A28–B35 were more common in infants with late-pattern (perinatal/acquired) BA [127]. Few other studies failed to confirm any significant differences in the distribution of any of the *HLA* genes tested comparing patients and controls [128,129].

Polymorphism of the *VEGF* gene and susceptibility to BA has also been investigated. The *VEGF* +936 C/T polymorphisms, particularly the C allele, are associated with BA [130]. One recent study investigated association of hepatic dysfunction with mutations in mitochondrial DNA (mtDNA) in BA [131]. Utilizing next-generation sequencing, mtDNA protein-coding genes were sequenced in liver specimens of 14 BA patients and 5 patients with choledochal cysts. Thirty four common nonsynonymous variations in mtDNA protein-coding genes were identified in all patients. Several SNP-like mutations in critical regions of complexes I to V were identified. These are involved in subunit assembly, proton-pumping activity and/or super complex formation in systematic 3D structural analysis. Hepatic dysfunction and liver injury in BA patients were also significantly correlated with the extent of hepatic failure, suggesting that the mtDNA mutations may aggravate hepatopathy. Recently, Tian *et al.* reported developing an *in vitro* human BA model, based on disease-specific induced pluripotent stem cells (iPSCs) generated from 6BA patients. They used nonintegrating episomal plasmids and determined that BA-specific iPSCs result in defective biliary differentiation [132].

## Vascular abnormalities

The bile duct system receives its blood supply exclusively from the hepatic artery and reduction in blood flow may lead to ischemic necrosis and fibrous obliteration of the bile ducts. Considering this, it has been hypothesized that primary vascular anomalies participate in the etiopathogenesis of BA. Ho *et al.* analyzed 11 cases of BA and reported hyperplastic and hypertrophic tortuous hepatic artery branches in both the extrahepatic and intrahepatic locations in all patients [133]. dos Santos *et al.* reported significant hypertrophy of hepatic arteries at the portoenterostomy site in infants with BA, when compared with infants without liver disease. They also recorded increased medial layer thickening when the portoenterostomy specimens were compared with the liver explants [134].

### Microchimerism

Maternal microchimerism has been suggested to play a role in the pathogenesis of BA, as well as certain autoimmune diseases [135]. Suskind *et al.* demonstrated an increased number of maternal cells in the livers of patients with BA compared with the livers of patients with neonatal hepatitis [136]. Muraji *et al.* quantitatively demonstrated higher numbers of XX+ cells in the sinusoids and portal tracts of male patients with BA [137]. Muraji hypothesized that the first hit is due to graft-versus-host disease by engrafted maternal effector T lymphocytes. They also suggested that the secondary effects that are manifested by progressive scarring are caused either by maternal chimeric effector T lymphocytes (e.g., graft-versus-host disease interaction) or targets (e.g., host-versus-graft disease interaction). Nijagal *et al.* reported improved allograft survival and retransplantation of liver in recipients with BA who received donor liver from maternal side, compared with those who received donor liver from paternal side, suggesting the possibility of immune tolerance secondary to exposure to noninherited maternal antigens [138]. Future research in this area is warranted and required.

## Future perspective

Investigators throughout the world agree that good clinical outcomes in BA are unlikely without a better understanding of the underlying etiology and pathophysiology of this disease. Future studies for environmental toxins and viruses may identify specific pathways for duct epithelial injury and fibrogenesis in BA. High-density immunophenotyping and cell sequencing of lymphomononuclear inflammatory cells in peripheral blood, liver and the extrahepatic bile ducts may identify cellular crosstalk and molecular targets to block mechanisms of disease. In-depth analysis of the data generated from genome-wide association studies and whole genome sequencing studies using next generation sequencing may provide novel insights into BA pathomechanism. New human BA models can be provided by disease-specific iPSCs for better understanding of the genetic basis of abnormal biliary tree development and opportunities to identify drugs that have therapeutic effects on BA.

**Executive summary**

- Biliary atresia is a complex disease of multifactorial aetiology that manifests as conjugated hyerbilirubininemia in neonates and infants. Biliary atresia has highly damaging consequences to infant health with rapid progression to portal hypertension and end-stage liver disease if not treated in a timely fashion. Despite Kasai portoenterostomy, the destructive bile duct injury progresses, leading to biliary cirrhosis in the majority of children.
- Morphologic examination of percutaneous liver biopsy tissue core is a key element in the diagnostic work-up of infants with suspected biliary atresia. Ducts/ductular bile plugs and portal stromal oedema are the strongest histologic predictors of large duct obstruction.
- Etiopathogenesis of biliary atresia is multifactorial and our understanding of the disease pathomechanism has evolved over the years. A variety of insults including viral infections, toxins, genetic variants, immune dysfunction, maternal microchimersim, vascular disturbances and abnormal morphogenesis have been postulated leading to biliary injury and obstruction.
